# Materials for Heavy Metals Removal from Waters

**DOI:** 10.3390/ma17091935

**Published:** 2024-04-23

**Authors:** Magdalena Balintova, Adriana Estokova

**Affiliations:** 1 Department of Environmental Engineering, Institute for Sustainable and Circular Construction, Faculty of Civil Engineering, Technical University of Kosice, Vysokoskolska 4, 042 00 Kosice, Slovakia; 2 Department of Material Engineering, Institute for Sustainable and Circular Construction, Faculty of Civil Engineering, Technical University of Kosice, Vysokoskolska 4, 042 00 Kosice, Slovakia; adriana.estokova@tuke.sk

Although heavy metal ions are naturally present in the environment, their concentrations have significantly increased due to industrial activities. Waste and wastewater, primary sources of heavy metals, necessitate treatment to immobilize pollutants or convert them into less harmful forms. Current research is predominantly focused on developing efficient treatment technologies that employ physicochemical, electrochemical, or oxidative processes. Among these methods, adsorption represents an ideal solution for effectively removing dissolved pollutants from contaminated streams using suitable adsorbents.

Recent studies have extensively explored the adsorption of pollutants from water using diverse materials with organic, biological, or mineral origins, such as agricultural waste, biomass from aquatic and terrestrial sources, biochar from soil and mineral deposits, and other locally available waste materials. Adsorption has emerged as the preferred technology for water purification due to its operational simplicity, cost-effectiveness, and minimal reliance on complex infrastructure. Additionally, it generates no additional toxic by-products, and low-cost sorbents can be used either in their natural state or after appropriate modifications to enhance their adsorption capacity.

This Special Issue, which highlights innovative trends in heavy metal removal utilizing advanced materials, reagents, and technologies that align with environmental and economic requirements, serves as a platform for scientists to showcase their original research on “Materials for the Removal of Heavy Metals from Water”.

This Special Issue, which contains nine original research papers from different countries, highlights recent progress in the removal of heavy metals from waters using various materials as adsorbents. We provide a brief overview of each paper, emphasizing the progress made in this critical field.

Biosorption, utilizing either live or dead microorganisms, has emerged as a cost-effective technique for wastewater treatment. Zinicovscaia and co-authors [1] investigated the applicability of yeast cells of Saccharomyces cerevisiae (S. cerevisiae) across four batch systems: Zn(II), Zn(II)-Sr(II)-Cu(II), Zn(II)-Ni(II)-Cu(II), and Zn(II)-Sr(II)-Cu(II)-Ba(II), as well as a real effluent sample. Synthetic effluents were modeled based on data obtained from real galvanic processes, while the real effluent, containing zinc and other metal ions in varying concentrations, was sourced from an electroplating company in Dubna, Russia. The study investigated the influence of different parameters on metal removal from the batch systems, with pH emerging as a significant factor affecting metal removal by biological sorbents. Optimal metal ion sorption occurred within a pH range of 3.0–6.0. Across the studied systems, the removal of Zn(II) ranged from 85% to 100%, with the highest efficiency observed in the Zn(II) system. The introduction of other metal ions led to a slight reduction in Zn(II) removal by S. cerevisiae biomass.

Surface adsorption, chemisorption, and ion exchange were identified as the main mechanisms of metal removal from the solution, with various functional groups, such as OH, C=C, C=O, C–H, C–N, and NH, participating in biosorption. By adjusting the pH and sorbent concentration to 10 g/L, it was possible to reduce the concentrations of Cu(II), Ba(II), Sr(II), and Ni(II) below the maximum permissible levels. The study demonstrated that the highest efficiency in removing Zn(II) ions from the real complex effluent was achieved using a subsequent two-stage system with the addition of a new biosorbent (20 g/L of biosorbent in the first stage and 10 g/L in the second stage) operating at pH 6.0 with a contact time of 60 min. Overall, the yeast *S. cerevisiae* proved to be an excellent candidate for treating zinc-containing complex effluents.

Despite the exceptional adsorption properties and outstanding performance of various biomass-based biosorbents, challenges still hinder the commercialization of biosorption. One such obstacle is the complex separation of spent biosorbents from water bodies, limiting their recyclability. Magnetic separation, a renewable recycling technology, offers higher efficiency compared to other separation methods (e.g., sedimentation, filtration) and is commonly utilized in water purification. In [2], a magnetically responsive biocomposite derived from *Rhytidiadelphus squarrosus* (*R. squarrosus*) was successfully developed using microwave-synthesized nanoparticles and microparticles of iron oxides. This biocomposite was then applied for the efficient removal of Co^2+^ and thioflavin T (TT) from aqueous solutions. The biocomposite exhibited enhanced biosorption performance compared to its non-magnetic biomass counterpart. Experimental data for both Co^2+^ and TT biosorption fitted well with the pseudo-n-order kinetic model and Langmuir isotherm, with maximum adsorption capacities of 218 μmol g^−1^ for Co and 483 μmol g^−1^ for TT. EDX elemental mapping revealed the presence of oxygen-containing functional groups from biomass biopolymers, along with synergistic interactions with –FeOH groups of iron oxides, contributing to Co^2+^ and TT adsorption. Cycling experiments demonstrated that the biocomposite retained 75% of its adsorption capacity for TT and 55% for Co^2+^ after the fourth cycle, indicating high adsorption efficiency and good reusability, particularly for TT removal. Based on these results, the prepared magnetic biosorbent outperforms other adsorbents due to its simple preparation, efficient separation, reusability, and high stability. This makes it suitable for both dye and metal adsorption from contaminated effluents and wastewater streams.

Frišták and co-authors [3] conducted physicochemical characterization of cherry pit biochar (CPB) produced from cherry pit biomass (CP) at 500 °C and evaluated its arsenic (As) and mercury (Hg) sorption capabilities in aqueous solutions, comparing them to those of activated carbon (AC). AsO4^3−^ anions and Hg^2+^ cations served as model contaminants to assess the sorption properties of the materials. CPB exhibited a higher sorption efficiency for the cationic form of Hg compared to the anionic As. Notably, CPB demonstrated excellent Hg sorption results, achieving approximately 43% sorption, whereas the sorption performance of As was relatively lower. The sorption of Hg was influenced by the concentration of phenolic, hydroxyl, and carboxyl functional groups on the sorption surfaces. Interestingly, CPB produced at 600 °C exhibited reduced sorption capacity compared to CPB produced at 300 °C due to the loss of oxygen-rich functional groups. These findings underscore the significance of oxygen-containing functional groups on the biochar surface in As sorption. Arsenic anions interacted with positively charged functional groups through electrostatic attraction, with biochar exhibiting higher protonation of functional groups at lower pH levels. CPB derived from cherry pits exhibited notable potential in sorbing Hg from aqueous environments, with sorption efficiency ranking in the order of CP < CPB < AC. Conversely, As sorption efficiency was markedly lower across all tested sorbents, with an order of CPB < CP < AC in terms of sorption success. The sorption rates were notably influenced by pH and the concentration of surface functional groups.

Wooden sawdust, a by-product of the wood industry, is one of the most widely available and cost-effective materials for removing pollutants from wastewater. Kovacova and co-authors [4] investigated the efficacy of alkali-modified sawdust (derived from poplar, cherry, spruce, and hornbeam) for metal removal across varying initial concentrations of Cu(II) and Zn(II) in model solutions. Their study revealed that poplar sawdust modified with KOH exhibited the highest adsorption efficiency, with copper removal reaching 94.3% at pH 6.8 and zinc removal reaching 98.2% at pH 7.3. Across all types of sawdust, it was observed that the sorption efficiency of modified sorbents surpassed that of untreated sawdust. The pH values initially increased more significantly with modified sawdust (for example, a value of 8.2 was achieved for zinc removal with spruce modified by NaOH) before gradually decreasing (to 7.0 for Zn(II) with spruce modified by NaOH). FTIR spectral analysis confirmed an augmentation in the number of –OH functional groups for both NaOH- and KOH-modified sawdust. The isotherm data revealed a superior fit with the Langmuir adsorption model compared to the Freundlich model, particularly in predicting the monolayer adsorption capacities of copper and zinc by both natural and modified wooden sawdusts. The ion exchange or hydrogen binding mechanism offered a plausible explanation for the adsorption of zinc and copper by wooden sawdust. Notably, the highest removal efficiency in the solution was attained at a concentration of 10 mg/L for both copper and zinc.

In another study, Mandal and co-authors [5] successfully developed a novel nano-biocomposite (CS-KAC-Ag) by embedding silver nanoparticles within kenaf-based activated carbon in the presence of a chitosan matrix using a straightforward photoirradiation method for removing cadmium ions from drinking and groundwater. The BET value of the nano-biocomposite was measured at 204 m^2^/g. The synthesized material exhibited high adsorption efficiency for the purification of mining wastewater containing Co^2+^, Cr^6+^, and Ni^2+^. This innovative, cost-effective, and sustainable nano-adsorbent was able to eliminate 95.1% of Cd^2+^ under optimal conditions, followed by Cr^6+^ (91.7%), Ni^2+^ (84.4%), and Co^2+^ (80.5%). The optimal parameters for pH, contact time, and adsorbent dose were found to be 9.0, 120 min, and 20 mg, respectively. The nano-adsorbent displayed excellent agreement with the pseudo-second-order model, with an R2 value exceeding 0.99. In addition, the porous adsorbent maintained its metal removal efficiency during four consecutive adsorption/desorption cycles. The developed CS-KAC-Ag material represents a sustainable, cost-efficient, and reusable solution for the remediation of toxic metal ions from wastewater. Additionally, this novel adsorbent holds promise for the removal of organic and microbial pollutants from wastewater streams.

In [6], the authors present a novel application of abandoned calcined mussel shells, where waste mussel shells underwent calcination to produce a sorbent material. Characterization of the shell powder before and after calcination revealed a transformation from calcium carbonate to calcium oxide, accompanied by the development of a porous surface structure. Using Pb(II) as a representative contaminant, the study investigated various factors influencing adsorption and analyzed the adsorption mechanism. The findings indicated that the adsorption behavior aligns with the Freundlich adsorption isotherm and the pseudo-second-order model. The calcined mussel shell powder demonstrated remarkable adsorption capacity for Pb(II), reaching 102.04 mg/g. Prior to calcination, the adsorption capacity stood at 32.34 mg/g, whereas post-calcination, this value increased, suggesting that calcination effectively enhances the adsorption performance of the shell powder towards Pb(II). This enhancement may be attributed to the decomposition of calcium carbonate into calcium oxide during calcination, facilitating the release of carbon dioxide and the creation of numerous small micropores. These observations were further supported by the results obtained upon characterization of the shell powder before and after calcination.

Zinicovscaia and co-authors [7] tested the capacity of a mineral–organic hybrid adsorbent, consisting of a *Shewanella xiamenensis* biofilm and zeolite (clinoptilolite of the Chola deposit), to remove metal ions from nickel-containing batch systems under different experimental conditions. In synthetic effluents with different chemical compositions, the hybrid adsorbent showed a high affinity for cations, while anion adsorption did not occur due to the negative charge of the adsorbent surface at all studied pH values. The process of cation sorption was shown to be pH-dependent, and the optimal pH for maximum metal ion removal (>70%) was 6.0. The experimental values at equilibrium were better adjusted to the Langmuir and Temkin isotherm models. The good fit of the pseudo-second-order and Elovich models suggested that chemisorption and ion exchange were the main mechanisms of metal sorption. The thermodynamic data showed that the biosorption process was spontaneous and endothermic. An increase in biosorbent dosage from 0.5 to 2.0 g did not significantly affect Ni(II) removal from a real industrial effluent, and it constituted 26%. However, a 12-fold dilution resulted in the removal of 72% of Ni(II) at a sorbent dosage of 1.0 g. The produced hybrid adsorbent can be considered to be a sorbent with a high affinity for metal cations and can be utilized for the treatment of diluted complex industrial effluents.

In [8], the authors carried out a detailed investigation that focused on the removal of nickel through adsorption onto natural bentonites sourced from four distinct Slovak deposits: Lieskovec (L), Hliník nad Hronom (S), Jelšový Potok (JP), and Stará Kremnička (SK). Employing batch processing conditions, the experiments varied the concentration of Ni(II), temperature, and time. The adsorption process exhibited rapid kinetics, nearing equilibrium within 30 min. The Langmuir maximum adsorption capacities for the four bentonite samples were determined to be 8.41, 12.24, 21.79, and 21.93 mg·g^−1^, respectively. Notably, the results were best fitted by the pseudo-second-order kinetic model, with constant rates ranging from 0.0948 to 0.3153 g·mg^−1^ min. Furthermore, among the bentonites used, the maximum adsorption capacity followed the order of SK > JP > L > S.

Arsenic stands out globally as one of the most prevalent contaminants in groundwater, posing significant toxicity concerns. Its most common forms in water include arsenite (in oxidation state +3) and arsenate (in oxidation state +5). In a pioneering study by Kazantsev and colleagues [9], the adsorption properties of nanostructured AlOOH/FeAl_2_ composites were investigated for the first time. These composites were prepared through the oxidation of bimetallic Al/Fe nanoparticles with varying iron contents. Upon oxidation, boehmite AlOOH nanosheets were formed, with the resulting FeAl_2_ nanoparticles distributed on the surface of these nanosheets. The adsorption capacity of the nanostructures derived from Al/Fe nanoparticles containing 10 wt.% Fe was measured at 112 mg/g of As. This capacity increased to 248 mg/g for nanostructures containing 20 wt.% Fe and remained at 240 mg/g for those containing 30 wt.% Fe. However, there was no corresponding increase in sorption capacity for As(V) species with nanostructures prepared from Al/Fe nanoparticles containing 30 wt.% Fe, possibly due to the enlargement of FeAl_2_ intermetallic particles. Consequently, the optimal ratio of Al to Fe in the Al/Fe nanoparticle composition was determined to achieve an AlOOH/FeAl_2_ composite with high adsorption capacity towards As(V). These findings hold significant promise for addressing the environmental challenges associated with arsenic removal from water sources.
